# Class I PI3K Provide Lipid Substrate in T Cell Autophagy Through Linked Activity of Inositol Phosphatases

**DOI:** 10.3389/fcell.2021.709398

**Published:** 2021-08-12

**Authors:** Ian X. McLeod, Ruchi Saxena, Zachary Carico, You-Wen He

**Affiliations:** Department of Immunology, Duke University Medical Center, Durham, NC, United States

**Keywords:** autophagy, PI3K I, T cells, lipid kinase, lipid phosphatase, cytokines, HIV

## Abstract

Autophagy, a highly conserved intracellular process, has been identified as a novel mechanism regulating T lymphocyte homeostasis. Herein, we demonstrate that both starvation- and T cell receptor-mediated autophagy induction requires class I phosphatidylinositol-3 kinases to produce PI(3)P. In contrast, common gamma chain cytokines are suppressors of autophagy despite their ability to activate the PI3K pathway. T cells lacking the PI3KI regulatory subunits, p85 and p55, were almost completely unable to activate TCR-mediated autophagy and had concurrent defects in PI(3)P production. Additionally, T lymphocytes upregulate polyinositol phosphatases in response to autophagic stimuli, and the activity of the inositol phosphatases Inpp4 and SHIP are required for TCR-mediated autophagy induction. Addition of exogenous PI(3,4)P_2_ can supplement cellular PI(3)P and accelerate the outcome of activation-induced autophagy. TCR-mediated autophagy also requires internalization of the TCR complex, suggesting that this kinase/phosphatase activity is localized in internalized vesicles. Finally, HIV-induced bystander CD4^+^ T cell autophagy is dependent upon PI3KI. Overall, our data elucidate an important pathway linking TCR activation to autophagy, via induction of PI3KI activity and inositol phosphatase upregulation to produce PI(3)P.

## Introduction

Autophagy is a major pro-survival mechanism, involved in intracellular remodeling and delivery of membrane-bound structures, termed autophagosomes, to the lysosome for turnover of the encompassed components into constituent building blocks (Bento et al., 2016). The autophagosome goes through a maturation process, reminiscent of late endosomal maturation, whereby the compartment becomes acidified when it fuses with lysosomes, during which further substrates can be imported for degradation, through the activity of adaptor molecules and autophagosomal receptors (Deter, 1975). Autophagy plays an important role in orchestrating innate and adaptive immune responses (Schmid and Munz, 2007; Cui et al., 2019). It plays specialized functions in antigen processing and presentation (Münz, 2016; Øynebråten, 2020), and thymic education of CD4^+^CD8^+^ double positive thymocytes (Nedjic et al., 2008a,b). In lymphoid-lineage cells, autophagy plays homeostatic roles (Bronietzki et al., 2015), such as control of mitochondria levels and related gene functions (Miller et al., 2008; Pua et al., 2009) and ER membrane trimming (Jia et al., 2011) and regulation of cytokine production (Galle-Treger et al., 2020; Singh et al., 2020).

In most systems studied, the autophagy induction machinery utilizes the coordinated activity of the class III PI3K complex, containing Vps34, to produce PI(3)P (Petiot et al., 2000). PI(3)P is a signal for the recruitment of effectors with FYVE- or GLUE-domains, in order to flex and fuse membranes (Nascimbeni et al., 2017), as well as an important signal in inward vesiculation and multi-vesicular body (MVB) formation (Zeng et al., 2006). In yeast, it has been shown that PI(3)P is imported into the autophagosome during autophagy (Axe et al., 2008). Vps34 has been shown to directly produce PI(3)P from phosphatidylinsotiol (PI), and has been demonstrated in several systems to be the major PI3K responsible for the production of cellular PI(3)P for autophagy (Yang and Van Kaer, 2020). T cells in which Vps34 was deleted entirely had slightly defective autophagy (Willinger and Flavell, 2012). However, *Vps34* null kinase domain knockout T cells have intact autophagy (Zhou et al., 2010; McLeod et al., 2011). Importantly, autophagy is regulated by general PI3K inhibitors, wortmannin, and 3-methyladenine (3MA) (Li et al., 2006; Pua et al., 2007; McLeod et al., 2011) and other classes of PI3K are also involved in the production of PI(3)P and the progression of autophagy in T lymphocytes (Bilanges et al., 2019). Additionally, since other classes of PI3K, especially class Ia isoforms, are activated during TCR engagement with peptide-MHC complexes (von Willebrand et al., 1994), we hypothesized these kinases are responsible for linking TCR activation to autophagy. Additionally, it has also been shown that Class I PI3K regulates autophagy by modulating protein synthesis and the Beclin 1 signaling pathway in malignant blood cells (Wang et al., 2017). The class I PI3K, P110β, functions as a positive regulator of autophagy by serving as a scaffolding protein (Dou et al., 2010, 2013). In contrast, knockdown of the class I PI3K, P110δ, in myeloma cells results in the potent activation of autophagy (Ikeda et al., 2010). Clearly, different isoforms of the class I PI3K have varying functions (Yu et al., 2015). This could be due in part to varying subcellular localizations or differing upstream receptors and adaptors.

Various inositol phosphatases are present in metazoan cells to modify the products of inositol kinases. The 3′ inositol phosphatase, PTEN, is responsible for the silencing of PI3K signals during TCR activation (Aquila et al., 2020), and enforces the need for costimulation through CD28 (Buckler et al., 2006). Knockout of another 3′ phosphatase, *Jumpy*, led to decreased autophagic flux (Vergne et al., 2009). Therefore, 3′ inositol phosphatases are important regulators of autophagy (Vergne and Deretic, 2010). However, the production of PI(3)P is likely influenced by other inositol phosphatases. 4′ phosphatases, including Inpp4a and Inpp4b can modify PI(3,4,5)P_3_, and are directly linked to PI3K activity by virtue of being in the same protein complex in human platelets (Munday et al., 1999). This complex is necessary for platelet aggregation through the coordinated activity of PI3KI and Inpp4 (Munday et al., 1999). Additionally, 5′ inositol polyphosphatases, including SHIP1 and SHIP2, are important in the modification of TCR induced signals, by dephosphorylating PI(3,4,5)P_3_ to PI(3,4)P_2_, and can form a complex with p85, the regulatory subunit of class I PI3K, and directly modify its activity (Jackson et al., 1995). The increased expression of SHIP was also shown to correlate with increased autophagy (Ngoh et al., 2015). It is heretofore unknown whether 4′ and 5′ inositol phosphatases affect autophagy induction and progression. In this study, we show that class I PI3K catalytic activity is essential for the induction of autophagy in T lymphocytes. We demonstrate that 4′ and 5′ inositol phosphatase activity is also required for autophagy induction by TCR stimulation, and that addition of PI(3,4)P_2_ can modify autophagy induction and progression. Additionally, we show that IL-7 inhibits autophagy in T cells, despite activating the PI3K pathway, while downregulating the transcription of Inpp4 and SHIP mRNAs. Finally, we demonstrate that HIV glycoprotein induced autophagy in human lymphocytes is dependent upon class I PI3K activity.

## Materials and Methods

### Mice

P85 conditional knockout mice (Luo et al., 2005) were purchased from the Jackson Laboratory, and crossed to ER-cre mice (Hayashi and McMahon, 2002) (The Jackson Laboratory). Genomic deletion of p85 was assessed by PCR primers detecting the floxed (1,275 bp) and deleted (298 bp) alleles (forward, GGT TTC TTA CTT TAG ACG GAG CTG; reverse, CCA GTT ACT TTC AAA TCA GCA CAG). Constitutively active P110α, mice (Srinivasan et al., 2009) were purchased from the Jackson Laboratory. Vps34-deficient T lymphocytes were generated by crossing Vps34-floxed mice (Zhou et al., 2010) to Lck-Cre transgenic mice (The Jackson Laboratory). All mice were bred and housed in Duke’s specific pathogen-free facilities in accordance with IACUC regulations.

### Human Samples

Human CD4^+^ T cells were isolated from the blood of healthy volunteers according to protocol approved by Duke’s Institutional Review Board. PBMC’s were isolated from whole blood using Ficoll-Paque Plus (GE Healthcare Bio-Sciences AB) according to the manufacturer’s instructions.

### Antibodies and Reagents

FITC, PE, PE-cy5, APC, APC-cy7, or Pacific Blue conjugated anti-CD3, -CD4, -CD8, -CD44, and -CD62L, were purchased from BioLegend, eBioscience, and BD Pharmingen. Anti-LC3 (PD015) and -p62 were purchased from MBL. Anti-PI(3)P, -PI(3,4)P_2_, -PI(3,4,5)P_3_, and PI(3,4)P_2_ lipid were purchased from Echelon Biosciences. IL-7, IL-4, and IL-15 were purchased from PeproTech. Acridine orange was purchased from Sigma. CytoID was purchased from Enzo Life Sciences. PIK75 was purchased from Cayman Chemical. iSHIP (AS1949490) and Dynasore were purchased from Tocris.

### Flow Cytometry

Single cell suspensions with RBC’s lysed were incubated with FcR blocker (2.4G2; eBioscience) and were immunostained for all surface markers. For intracellular staining, cells were fixed in 4% PFA for 20 min, washed, and permeabilized in 0.1% saponin for 20 min. All stains were performed in 0.1% saponin thereafter. Acridine Orange and CytoID were used at 0.1 μg/mL for 20 min at RT just prior to FACS analysis. All FACS utilized a BD Facscanto II (BD).

### Fluorescence Microscopy

All images were captured with a custom-built Zeiss Observer D1 using a Zeiss 100x objective lens and a 1.4 NA. Images were captured using a Photometrics CoolSNAP HQ2 and analyzed using Metamorph software for punctae number, size, and intensity. Images were deconvoluted and thresholded using Autoquant X2 software. Deconvolution was done blind at 40 iterations. LC3 punctate structures were defined as at least 10 pixels in size with fluorescence intensities of at least twice that of average background intensities. Flurochromes used included Pacific Blue, cy5, cy3, and FITC.

### siRNA Knockdown

Clones were purchased from Dharmacon. Pools of siRNA clones for Inpp4A were GCCGAGAGGUUUGGCGAUA, CAUC AUAGGUUGCAUUUAA, GAUCGAAAGCCAAAUAGUU, and GAUGAGAGUUCAAGACGAU. Pools for Inpp4B were ACG AGAACAUUACGUGGUA, GAAGGAUUGUUAAGUACAU, A AUGAUGUAUUGCCAGUUA, and CGAUGAAAUUGGAAU GUUA. Pools for SHIP1 were CGACAGGGAUGAAGUACAA, GAAUUGCGUUUACAUUAC, GCAUUGCCCUUCGGUUAG A, and UGACAGCGACGAAUCCUAU. Pools for SHIP2 were UCAAGGAGCUUACGGAUCU, GUCAGUACGUCCAGUGU GA, CCAAGAAAGGGCUCUCAAA, and GCACACGUAUCGC AUUCUG. siRNA clones were electroporated into freshly isolated human peripheral blood mononuclear cells (hPBMC’s) and allowed to recover for 3 days before treatments for AVO formation. Primers used to detect knockdown efficiency included TAAGCTGAGGAACTGCCTGCATGA and TGGA AGTGGCCTGAGTGACTTTGA for Inpp4A, GTGGCGGCAA CAATGATGGAGAAA and TACGCAAGTTCCTGAAGGAGCA CA for Inpp4B, TCGGGACAAATACGCCTACACCAA and TGGGAAGTGACTCCTGCCTCAAAT for SHIP1, and TAA GTCCCAGCGTGTCCAGAACAA and TTCCCATGTTCCAG GTGCCTATGA for SHIP2.

### Statistical Analysis

Graph Pad Prism was used for statistical analysis and the differences between groups was calculated using Student’s *t*-test. *p*-values < 0.05 were considered statistically significant.

## Results

### Class I PI3K Activity Is Required for T Cell Starvation-Induced Autophagy

**FIGURE 1:**
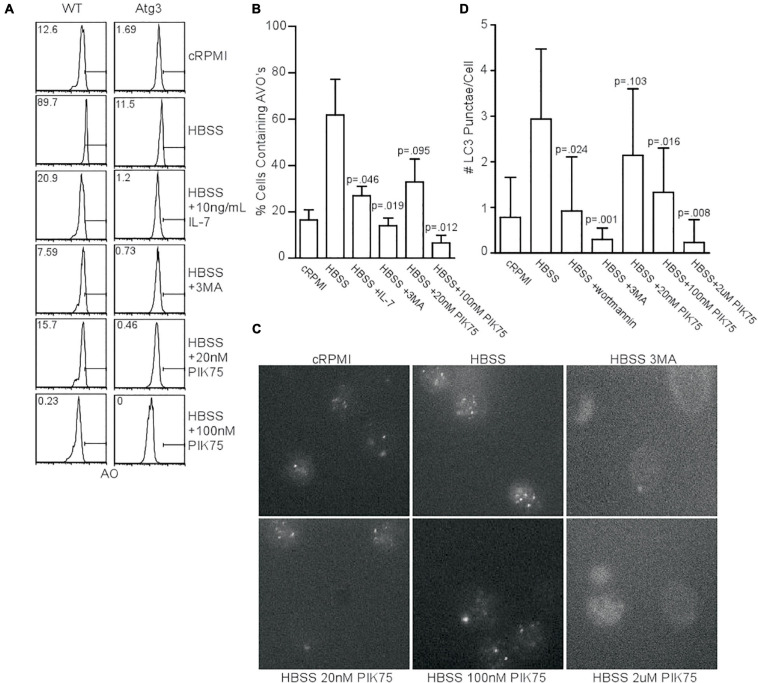
Class 1 PI3K activity is necessary for starvation-induced autophagy. **(A)** AVO profiles gated on live CD4 T lymphocytes starved for 48 h in HBSS and treated with the indicated activators inhibitors of PI3K. A 5mM 3MA and higher doses of PIK75 drastically inhibited AVO formation, while 20 nM PIK75 and 10 ng/mL IL-7 had slightly less potent effects. Atg3^*f*/f^Lck-cre T cells were used as a negative control to assess background lysosomal contribution. Gates and numbers indicate the percentage of cells that have upregulated autophagy above basal levels. **(B)** Quantitation of **(A)**, representative of at least 4 independent experiments per condition. **(C)** Fluorescence microscopy of endogenous LC3 punctate structures 48 h post starvation with the indicated inhibitors. **(D)** Quantitation of starvation-induced LC3 aggregates 48 h post starvation. Data are representative of at least 2 independent experiments per condition with at least 30 cells counted in each replicate.

Since kinase activity of the class III PI3K, Vps34, is dispensable for autophagy induction in T cells (McLeod et al., 2011), if not the entire protein itself (Willinger and Flavell, 2012), we investigated what other classes of PI3K were required for starvation-induced autophagy. We measured acidic vesicular organelle (AVO) formation in naïve T cells from WT mice with acridine orange staining to screen for compounds of interest. Starved mouse CD4^+^ T cells had increased AVOs, while the addition of interleukin 7 (IL-7), 3-methyladenine (3MA), and the class I PI3K inhibitor, PIK75, all inhibited autophagy (Figure 1A). The use of PIK75 at 20 nM reflected an IC_50_ for PI3KIα, while 100 nM reflects an IC_50_ of PI3KIγ and δ. A 2 μM PIK75 inhibits all four class I isoforms. Atg3^*f*/f^Lck-cre CD4^+^ T cells were used as a negative control, as they have severely impaired autophagy (Jia and He, 2011). The pan-PI3K inhibitor, 3MA, and 100 nM PIK75 had the greatest impact upon AVO formation (Figures 1A,B), suggesting that PI3Kγ and/or δ play important roles in starvation-induced autophagy in T cells.

We next sought to validate the AVO’s observed in Figure 1A were *de facto* autophagosomes using endogenous LC3II punctate formation as a measure of autophagy induction (McLeod et al., 2011). Starvation increased the number of LC3 structures, while 3MA, wortmannin, 100 nM PIK75, and 2 μM PIK75 reduced the number of LC3 structures (Figures 1C,D). A 20 nM PIK75 had a much smaller inhibitory effect on autophagy induction, suggesting that P110α is only minimally involved in starvation-induced autophagy in primary T cells (Figure 1D). To further investigate the role of P110α in autophagy induction, mouse T cells were induced to express a constitutively active form of P110α (P110ca^*f/f*^ER-cre). Forty eight hours h post-induction, CD4^+^ T cells had increased Akt activity, demonstrating a constitutively active PI3K pathway (Supplementary Figure 1A). Although P110ca expressing naïve T cells cultured in complete media had slightly increased levels of LC3^+^ punctae (Supplementary Figure 1B), no difference in AVO formation could be detected 48 h post-starvation (Supplementary Figures 1C,D), further suggesting that P110α is not the major isoform responsible for autophagy induction.

### TCR-Mediated Autophagy Requires Class I PI3K

**FIGURE 2:**
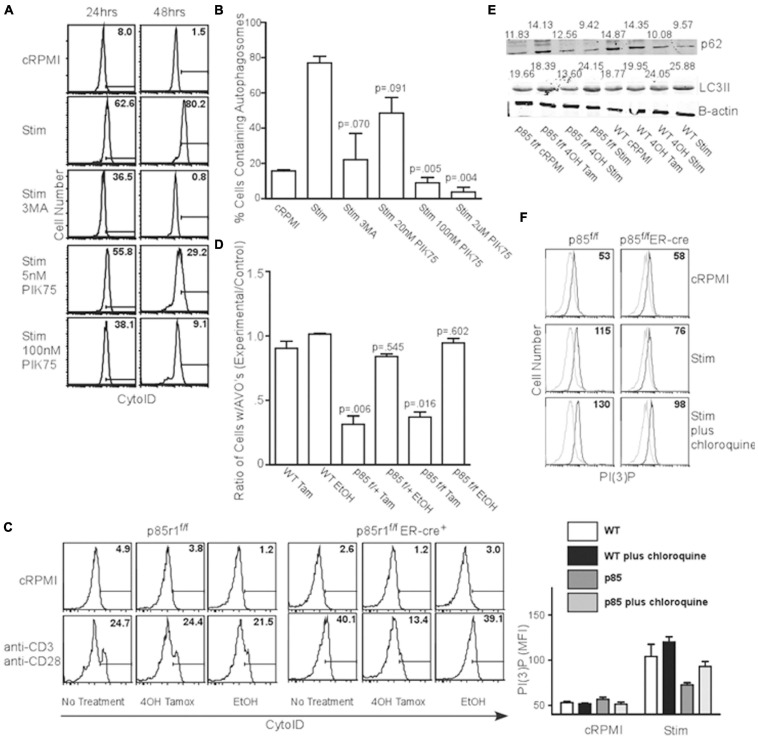
TCR-induced autophagy is dependent upon class I PI3K and 5′ phosphatase activity. **(A)** AVO formation histograms gated on live CD4 T lymphocytes stimulated with 1ug/mL soluble anti-CD3 and anti-CD28 for 24 and 48 h with the indicated reagents. A 5 mM 3MA, 100 nM PIK75, and 2 μM PIK75 potently reduced AVO formation, especially at 48 h. Histograms are representative of at least 3 independent experiments under each condition. **(B)** Quantitation of AVO formation from (A) summarizing at least 3 independent experiments per condition. **(C)** AVO formation in p85^*f*/f^ and p85^*f*/f^ER-cre CD4 T cells demonstrates the requirement for class I PI3K in TCR-mediated autophagy induction. Splenocyte and lymphocyte mixtures were cultured for 4 days in 1 ng/mL IL-7 and either 500 nM 4OH Tamoxifen or EtOH, and stimulated for 2 days with 1 μg/mL soluble anti-CD3 and anti-CD28 for 48 h. **(D)** Quantification of **(C)**. Ratios of AVO formation of CD4 T cells pre-treated with 4OH Tamoxifen or EtOH to those kept in complete media for 96 h. Data are compiled from 3 independent experiments. **(E)** p62 degradation and LC3 lipidation are impaired in p85-deficient CD4 T cells. Cells were treated as in **(C)**. Numbers indicate band densities compared to those of β-actin. **(F)** PI(3)P levels measured 24 h after activation through soluble anti-CD3 and anti-CD28 as in **(C)**.

T cell receptor (TCR) signaling is a potent activator of autophagy in T cells (Pua et al., 2007; Botbol et al., 2016). TCR engagement is known to activate the class I PI3K pathway, suggesting a link between the processes. To investigate this, we activated splenocytes with anti-CD3 and anti-CD28 for 24 or 48 h in the presence of various PI3K inhibitors, and examined AVO induction using CytoID, a cationic amphiphilic tracer dye specific to autophagosomes (Kauntz et al., 2011). TCR stimulation induced AVO formation, which was inhibited by 100 nM and 2 μM PIK75, suggesting p110β, γ, or δ isoforms are potentially involved in TCR-induced autophagy (Figures 2A,B). However, p110 kinase activity results in the production of PI(3,4,5)P_3_, with very little PI(3)P (Auger et al., 1989). However, PI(3)P is required for autophagy (Dall’Armi et al., 2013). This suggests the involvement of other enzymes related to phosphatidylinositol phosphate production. Two different inositol phosphatase families, with linked activity, would be necessary to derive PI(3)P from PI(3,4,5)P_3_. A 5′ inositol phosphatase, such as SHIP, known to be involved in TCR signal quenching, could generate PI(3,4)P_2_ for PI(3)P production.

In order to fully inhibit class IA PI3K, we crossed mice with loxP sites flanking the *p85* regulatory subunit of PI3KI (which also contains the p55 and p50 splice variants that act as regulatory subunits for most class IA PI3K) to an estrogen inducible *cre* recombinase to create *p85^*f*/f^ER-cre* mice (Vooijs et al., 2001; Luo et al., 2005). Ninety six hours after deletion with 4-OH Tamoxifen, all the floxed allele was absent, leaving only the deleted allele (Supplementary Figure 1E). These mice are deficient in all three p85a isoforms including full length p85a and the truncated proteins p55a and p50a (Luo et al., 2005; Vanhaesebroeck et al., 2005). When the TCR of *p85^*f*/f^ER-cre* or *p85^*f*/+^ER-cre* CD4^+^ T lymphocytes were stimulated, they showed a 60-80% impairment in AVO formation (Figures 2C,D). Additionally, stimulated *p85^*f*/f^ER-cre* T cells had a 50% reduction in LC3II processing from undeleted controls (Figure 2E lower panel, quantified in Supplementary Figure 2D). p85 and p55 deletion almost completely inhibited autophagic flux, as assessed by the degradation of p62, after TCR stimulation, whereas WT T cells had a 40% degradative efficiency (Figure 2E upper panel, quantified in Supplementary Figure 2C). Furthermore, p85-deficient T cells had a production of PI(3)P that was only half of WT controls 24 h post TCR stimulation (Figure 2F), though the lysosomal turnover of PI(3)P was not impaired, as chloroquine enhanced PI(3)P levels in both WT and p85-deficient T cells (Figure 2F).

However, T cells were still able to be fully activated, both after treatment with various concentrations of PIK75, and in the absence of p85 as assessed by the upregulation of activation markers CD44 and CD69, as well as growth of T cells into blasts (Supplementary Figures 2A,B). Only treatment with 3MA and the highest concentration of PIK75 has any discernible effect on T cell activation (Supplementary Figure 2A). These results show PI3K kinase activity is required for TCR-induced autophagy.

### Common γ_*c*_ Cytokines Inhibit Autophagy Induction

**FIGURE 3:**
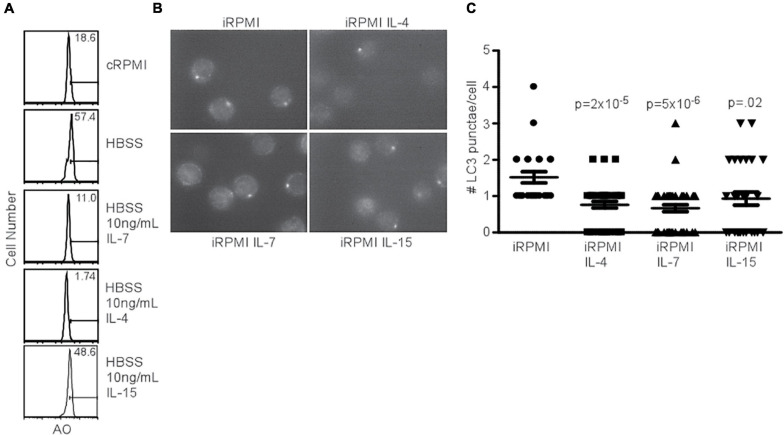
Common gamma chain cytokines inhibit starvation-induced autophagy. **(A)** AVO profiles of naïve CD4 T lymphocytes starved in HBSS for 48 h with the indicated cytokines. Histograms are representative of 3 independent experiments. IL-4 and IL-7 had potent effects on starvation-induced AVO formation, while IL-15 had a less drastic effect. **(B)** Fluorescence microscopy of LC3 aggregates in naïve, starved CD4 T lymphocytes treated with the indicated γ_*c*_ cytokines for 48 h, permeabilized, and stained with anti-LC3. Data are representative of fields observed. **(C)** Quantitation of **(B)**, with at least 30 cells counted for each condition in two independent experiments. The indicated cytokines significantly reduced LC3 punctate formation. *P*-values are from unpaired, two-tailed Student’s *t*-tests.

Since the addition of IL-7 was able to prevent AVO formation (Figure 1A), despite the ability of the γc chain to activate the PI3K pathway, we sought to determine the capacity of other homeostatic T cell cytokines to inhibit autophagy. IL-4 and IL-7 both had an inhibitory effect on AVO formation in CD4^+^ T cells, while IL-15 had a reduced potency (Figure 3A). The low inhibitory potential of IL15 compared to other cytokines could be due to low expression of its receptor CD122 on CD4^+^ T cells which is critical for binding and signaling of IL15 (Zhang et al., 1998; Keller et al., 2020). The capacity of IL-4, IL-7, and IL-15 to regulate T cell autophagy was confirmed using LC3 punctate formation (Figures 3B,C). Each cytokine was able to significantly reduce autophagy in naïve T lymphocytes. IL-4 and other Th2 cytokines have been previously shown to reduce autophagosomal delivery of *M. tuberculosis* to lysosomal compartments in macrophages (Harris et al., 2007). However, this paradigm can be extended to other homeostatic T cell cytokines. Though PI(3,4,5)P_3_ is generated during γ_*c*_ signaling and during TCR activation, the progression of autophagy from these two signals is remarkably different. This would suggest that additional factors are involved in mediating an autophagic response downstream of PI3K.

### Role of TCR Endocytosis in TCR-Induced Autophagy

**FIGURE 4:**
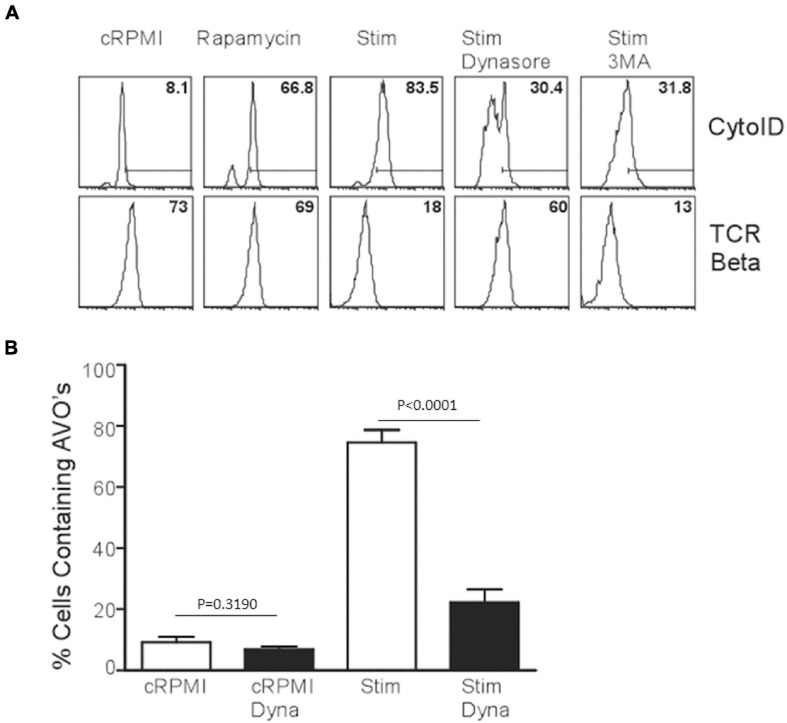
TCR internalization is required for TCR-mediated autophagy induction. **(A)** AVO formation in CD4 T cells pre-treated with 100 μM Dynasore or 5 mM 3MA for 1 h and stimulated for 24 h with 1 μg/mL soluble anti-CD3 and anti-CD28. Lower panel indicates the relative levels of TCR-β on the cell surface to demonstrate the efficacy of Dynasore versus other treatments. **(B)** Quantification of AVO formation in **(A)**, data represents 3 independent experiments.

Since p85 is required for the induction of autophagy in T lymphocytes, we hypothesized that the production of PI(3)P occurs on endomembranes after the internalization of the TCR complex. Subsequently, early endosomes are highly enriched for PI(3)P, and Vps34 is localized to early endosomes in T cells as well (McLeod et al., 2011). Utilizing dynasore to inhibit dynamin function, including the internalization of TCR complexes after TCR stimulation (Chaturvedi et al., 2011), AVO formation was examined. T cells treated with dynamin failed to upregulate AVO’s and had an almost complete block in TCR downregulation from the cell surface (Figures 4A,B), whereas rapamycin treated cells efficiently upregulated AVOs with no consequent TCR internalization, and 3MA treated cells had a block in AVO formation, but complete TCR internalization (Figures 4A,B). Hence, the signaling events required for TCR-induced autophagy induction occur post-internalization of the TCR complex on signaling endomembranes.

### SHIP 5′ Phosphatase Activity Is Required for TCR-Induced Autophagy

**FIGURE 5:**
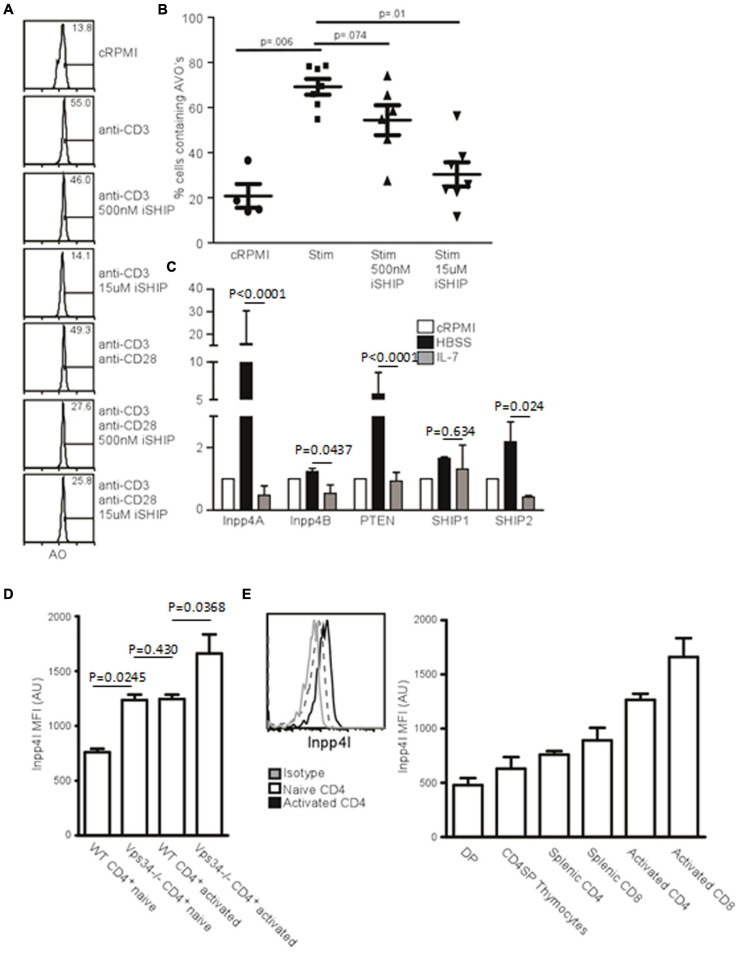
Inositol phosphatases respond to autophagic stimuli. **(A)** AVO formation in CD4 T lymphocytes pre-treated with 500 nM or 15 uM Anchor (AS1949490) for 1 h and stimulated with either 1 μg/mL soluble anti-CD3 alone or with anti-CD28 for 24 h. 500 nM Anchor inhibits SHIP2 activity and 15 μM inhibits both SHIP1 and SHIP2. **(B)** Quantification of AVO’s from **(A)**. *p*-values are from paired, two tailed, Student’s *t*-tests from 6 replicates in 3 independent experiments. **(C)** Relative levels of inositol phosphatase mRNA in CD4 T cells 48 h after starvation in HBSS or with 10 ng/mL IL-7. QPCR results were normalized to that of GAPDH. Data are representative of 3 independent experiments with 3 replicates per experiment. **(D)** Histogram of Inpp4A protein level in naïve or 24 h stimulated CD4 T cells, quantified in the right panel as 4 replicates from 3 independent experiments. Gray histogram represents the isotype control, the dotted histogram is the naïve level, and black histogram is the level in TCR activated cells. **(E)** Intracellular stains for Inpp4A protein levels in WT or Vps34^*f*/f^Lck-cre CD4 T cells (Vps34-/-). Data are from 3 replicates in 2 independent experiments.

As PI(3)P could be derived from PI(3,4,5)P_3_ catalyzed by SHIP in T cells, we tested the effect of pharmacological inhibition of SHIP on autophagy induction in T cells. The compound AS1949490 (Suwa et al., 2009), abbreviated iSHIP here, displayed inhibition of TCR-induced autophagy. We further sought to dissect the SHIP pathway by investigating the activity of both SHIP1 and SHIP2. Using 500 nM iSHIP, selectively inhibiting SHIP2 activity, and 15 μM iSHIP, inhibiting both SHIP2 and SHIP1, TCR-mediated AVO formation was measured. Although 500 nM iSHIP had only a minor impact on AVO formation, 15 μM almost completely inhibited any AVO upregulation (Figures 5A,B).

### Inpp4 Levels Are Regulated by Autophagic Stimuli

If SHIP-mediated hydrolysis of PI(3,4,5)P_3_ is required for the production of PI(3,4)P, a 4′ phosphatase activity would be required for the production of PI(3)P from PI(3,4)P_2_. Two 4′ phosphatases have been described, Inpp4A and Inpp4B (Dyson et al., 2012). We used quantitative PCR to measure how autophagic stimuli affect the levels of 4’ inositol phosphatases in T cells. IL-7 treatment decreased the levels of Inpp4A and Inpp4B, as well as the levels of SHIP1 and SHIP2 (Figure 5C). However, starvation had the opposite effect, with levels of Inpp4A increasing dramatically (Figure 5C). While Inpp4A is expressed at low levels by naïve T cells, TCR stimulation increases the level of Inpp4A twofold (Figure 5D). Additionally, naïve Vps34^*f*/f^Lck-cre T cells have increased Inpp4A and that level further increases upon TCR stimulation (Figure 5E). This could be a compensatory mechanism that allows further processing of PI(3,4)P_2_ into PI(3)P in the absence of Vps34. Hence the levels of various polyinositol phosphatases are positively regulated by autophagic stimulators and are negatively regulated by IL-7. This could account for the difference seen in the autophagic response between IL-7 treatment and TCR stimulation of T lymphocytes.

### PI(3,4)P_2_ Can Be Hydrolyzed to PI(3)P, Accelerating Autophagy

**FIGURE 6:**
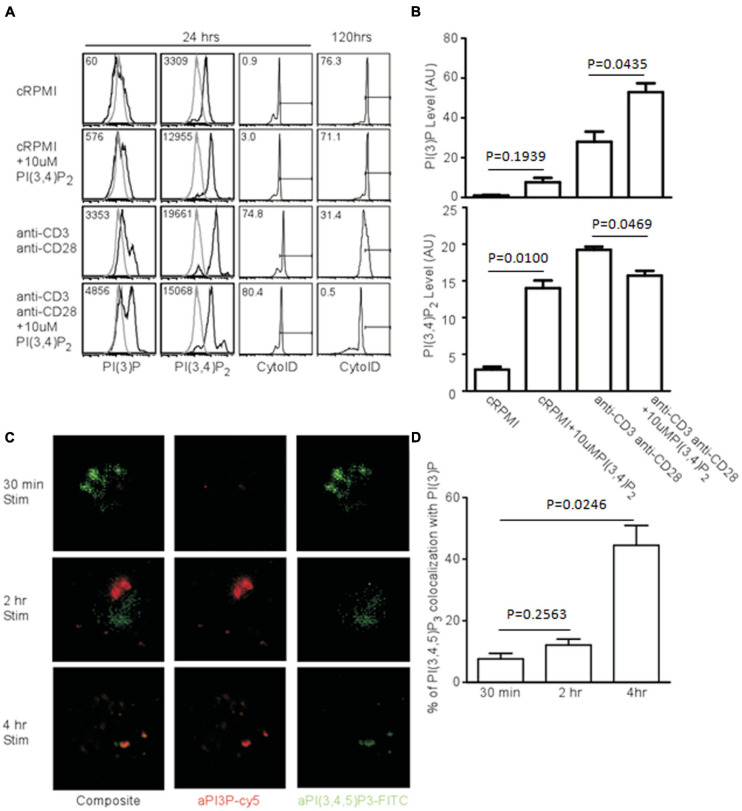
Exogenous PI(3,4)P_2_ alters the autophagic profile and is converted to PI(3)P. (**A**, left panel) Histograms of relative levels of PI(3)P and PI(3,4)P_2_ CD4 T cells were loaded with 10 μM PI(3,4)P_2_ and stimulated with 1 μg/mL soluble anti-CD3 and anti-CD28 for 24 and 120 h. PI(3,4)P_2_ is largely converted to PI(3)P only after TCR stimulation. (**A**, right panel) AVO formation of CD4 T cells loaded with PI(3,4)P_2_. Exogenous PI(3,4)P_2_ effects an accelerated autophagic profile that is resolved by 120 h compared to unloaded controls. **(B)** Quantification of phosphatidyl inositol levels from CD4 T cells left untreated or loaded with PI(3,4)P_2_ and stimulated for 24 h as in **(A)**. Data represent 4 replicates from 3 independent experiments. **(C)** Fluorescence microscopy of phosphatidylinositol species in T cells activated with plate-bound anti-CD3 and anti-CD28 for the indicated time periods. **(D)** Quantitation of colocalization of phosphatidylinositol species from **(C)**.

If PI(3,4)P_2_ is a relevant intermediate in TCR-mediated autophagy, the addition of exogenous phospholipids should impact the induction and progression of autophagy. To test this, we added PI(3,4)P_2_ to T cell cultures and measured both PI(3)P production and AVO formation. Naïve CD4^+^ T cells have very little PI(3)P and the addition of PI(3,4)P_2_ only slightly increased the amount of PI(3)P, despite the efficient loading of cells with PI(3,4)P_2_ (Figures 6A,B). However, TCR stimulation caused cells loaded with PI(3,4)P_2_ to produce twice the amount of PI(3)P and more efficiently hydrolyze PI(3,4)P_2_ (Figures 6A,B). This had a major impact on autophagic progression. Although by 24 h, AVO formation was only slightly enhanced (Figure 6A), 120 h later AVO’s had been completely turned over in stimulated T cells loaded with PI(3,4)P_2_, while TCR-stimulated cells in the absence of exogenous lipid were still undergoing some autophagy (Figure 6A right panels). Naïve T cells started to undergo autophagy after a few days, despite being kept in complete media culture conditions, possibly due to a stress response (Figure 6A). Consistent with our model, PI(3,4)P_2_ loaded naïve T cells did not upregulate autophagy over unloaded controls, since no upregulation of inositol phosphatases occurred (Figure 5C).

### PI(3)P Is Produced in PI(3,4,5)P_3_ Compartments

Since PI(3)P is produced from PI(3,4,5)P_3_ after TCR stimulation, this process might occur at distinct sites. To visualize this process, we stimulated naïve T cells. After 30 min, there was a huge production of PI(3,4,5)P_3_, but very little PI(3)P, with little compartmental overlap between the two species at 30 min and 2 h (Figures 6C,D). However, by 4 h, much of the PI(3,4,5)P_3_ had disappeared, and a significant proportion of what remained was bound up in PI(3)P-bearing vesicles, which were quite prominent at this time point (Figures 6C,D). Thus, PI(3)P accumulates in activated T cells contemporaneously with the quenching of PI(3,4,5)P_3_ in vesicles containing both phospholipids at a time point prior to the onset of autophagy.

### Human T Cells Undergo Autophagy Regulated by Class I PI3K and Inositol Phosphatases

**FIGURE 7:**
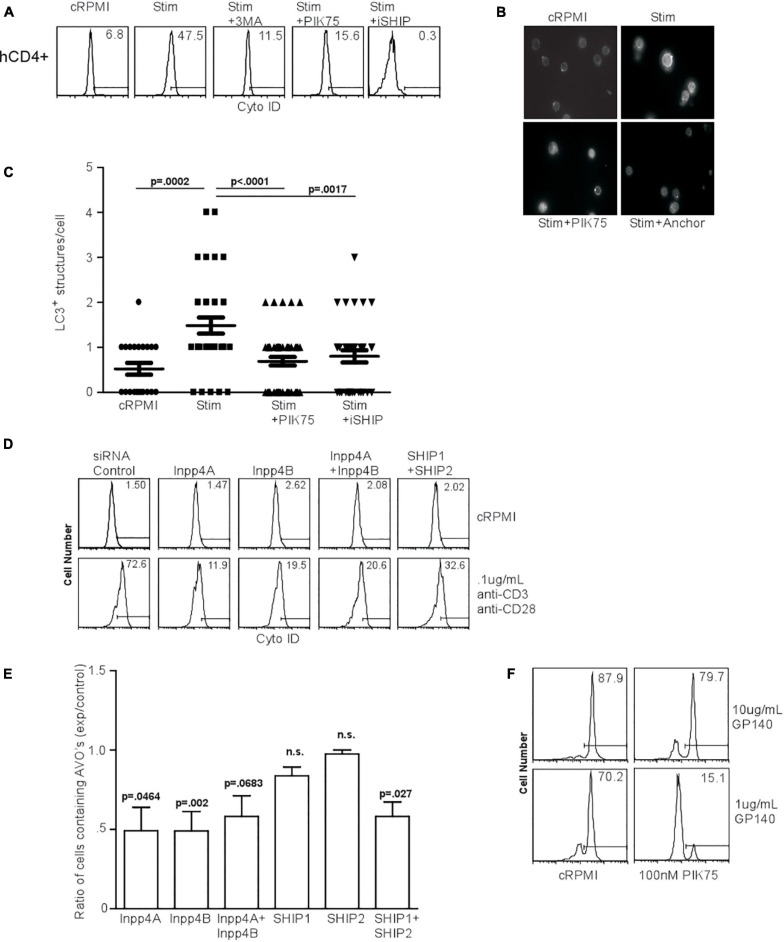
Human T cell autophagy is dependent upon class I PI3K activity and inositol phosphatase activity. **(A)** AVO formation in human lymphocytes. Human CD4^+^ T cells were isolated from healthy donors, purified by Ficol gradient centrifugation, and stimulated with 0.5 μg/mL anti-CD3 and anti-CD28 for 24 h. AVO formation was inhibited by 100 nM PIK75, 3MA, and 15 μM Anchor. Data are representative of at least 3 independent experiments per condition. **(B)** LC3 punctate formation in stimulated human CD4^+^ T cells for 48 h as in (A), permeabilized, and stained for endogenous LC3. **(C)** Quantification of **(B)**, representative of two replicates with at least 30 CD4 cells counted per condition. **(D)** AVO formation in siRNA mediated knockdown of Inpp4A, Inpp4B, and both SHIP1 and SHIP2. Human CD4^+^ T cells were purified as in **(A)** and electroporated to allow uptake of 5 μg/5 × 10^6^ cells siRNA cocktails, allowed to recover in complete media for 72 h and stimulated with 0.5 μg/mL anti-CD3 and anti-CD28 for 48 h. **(E)** Quantification of stimulated CD4 T cells in **(D)**, comparing the percentage of cells upregulating AVO formation in the siRNA knockdown samples to a scrambled control siRNA. Data are representative of cells from 5 donors in 4 independent experiments. **(F)** AVO formation in human CD4^+^ T cells exposed to soluble HIV GP140 fusion protein at the indicated concentrations. Total PBMC’s were treated with the glycoprotein and 100 nM PIK75 for 48 h.

HIV glycoprotein binding to CXCR4 and CD4 on human T lymphocytes has been proposed as a major mechanism by which uninfected, bystander cells die through autophagy, suggesting that CXCR4-mediated signaling induces autophagy in T cells (Espert et al., 2006; Espert et al., 2008). We first examined the effect of PI3K inhibitors on TCR-stimulation induced autophagy in human T cells. TCR stimulation-induced autophagy in human T cells was inhibited by 3MA, PIK75, and iSHIP (Figure 7A). To verify autophagy was initiated, we measured LC3 punctate formation in human T cells 48 h after TCR stimulation, and confirmed 100 nM PIK75 and iSHIP were able to ablate autophagy (Figures 7B,C). Furthermore, when either Inpp4A or Inpp4B were knocked down with siRNA cocktails, or when both SHIP1 and SHIP2 were knocked down (Supplementary Figures 3A,B), AVO formation via TCR stimulation was severely impaired (Figures 7D,E). SHIP1 and SHIP2 seemed to have some redundancy during this process, while Inpp4A and Inpp4B did not. However, the loss of both Inpp4 isoforms had no synergistic effect, suggesting they may function within the same complex. Thus, TCR-induced autophagy in human CD4 T cells is dependent on the coordinated activity of class I PI3K and inositol phosphatases.

### HIV Glycoprotein-Induced Autophagy Is Mediated by PI3K

The HIV glycoproteins GP40 and GP120 can bind to CXCR4 on uninfected T lymphocytes and induce autophagy (Espert et al., 2006). CXCR4 is a G-protein coupled receptor (GPCR), the downstream signaling of which often occurs through the class Ib PI3K, PI3Kγ (Suzuki et al., 2001). To test whether autophagy downstream of HIV glycoprotein binding was dependent on PI3K, we subjected human CD4 T cells to a soluble fusion protein consisting of the exodomains of GP41 and GP120, termed GP140. Co-culture of CD4 T cells with GP140 induced a high level of autophagy, which was sensitive to 100 nM PIK75 treatment, suggesting that HIV glycoprotein autophagy induction uses the same machinery as TCR-mediated autophagy induction (Figures 2, 7F).

## Discussion

The role of phosphatidyl-3 kinases has long been appreciated in pro-survival signaling (Shanware et al., 2013). Even as autophagy has vaulted to the forefront as a pro-survival mechanism, many aspects of its regulation remain unknown. Through yeast studies implicating Vps34 as the main proponent of PI(3)P production, and the use of various PI3K inhibitors, which we now know are non-specific, Vps34 has been assumed to be the main producer of cellular PI(3)P. However, studies from our lab showing Vps34 kinase activity is dispensable for autophagy induction in T lymphocytes, suggest that other PI3K classes important. The reduction in autophagy observed using specific PI3K inhibitors, and a knockout of p85/p55 in T cells, demonstrate that class I PI3K is the major player in autophagic activity. This was confirmed by observing autophagic flux in p85-deficient T cells. The specific isoform of PI3KI utilized by the cell is most likely dependent upon the nature of the autophagic stimulus. This could help explain the different degrees of inhibition observed when class Iα was pharmacologically deactivated with 20 nM PIK75 between starvation- and TCR-induced autophagy. Furthermore, we have not ruled out contributions of class II PI3K or class 1b PI3K on autophagy induction, as even the p85/p55/p50 knockout T cells still had a nominal level of autophagy.

Our study also investigated the requirement of inositol phosphatases to translate TCR signaling into autophagy. Using iSHIP, or by knocking down Inpp4 or SHIP, we demonstrated the necessity of these proteins to sustain PI(3)P production. Interestingly, both SHIP1 and SHIP2 had to be inhibited or removed before a significant reduction in autophagy could be observed, suggesting these proteins can compensate for one another. However, Inpp4A and Inpp4B could not compensate for one another, and knockdown of either one was sufficient to reduce autophagy. However, knockdown of both had no synergistic effect on AVO formation. This suggests that they operate within a single complex or pathway. The significance of the Inpp4 complex to participate in TCR-mediated autophagy was further confirmed by the ability of T cells to convert exogenous PI(3,4)P_2_ into PI(3)P, only after TCR stimulation, leading to a more rapidly resolved autophagy. This correlates very well with the upregulation of Inpp4A after TCR stimulation.

Indeed, the levels of all the polyinositol phosphatases correlated very well with autophagy. Starvation leads to the upregulation of Inpp4A, Inpp4B, SHIP1, and SHIP2, while IL-7 treatment leads to repression of all these phosphatases. The regulation of Inpp4A and SHIP2 are especially dynamic, suggesting that they might be the limiting factors in substrate conversion. It is the levels of these inositol phosphatases that we believe to account for the difference in autophagy observed in TCR-stimulated versus IL-7 treated T cells, despite the production in PI(3,4,5)P_3_ in both cases. This is important because pro-survival cues such as TCR stimulation and IL-7 treatment need to be quenched to avoid pathogenic T cell activation and proliferation. The activities of inositol phosphatases ensure that higher ordered species of inositol phosphates are not only removed, but also converted into intermediates for alternative pro-survival pathways such as autophagy. This paradigm goes beyond PI(3)P production for PI(3,4,5)P_3_, and can be applied to any membranes that need to be converted into another type of organelle membrane with an alternative phosphatidyl inositol coat (Botelho, 2009). We wish to stress that PI(3)P is a complex signal governing many functions. Though this signal is very low in naïve T cells (Figure 6A), TCR signaling produces much PI(3)P, of which only a fraction is ever bound for an autophagosome (Figures 6C,B). Much of this signal is undoubtedly used to degrade signaling molecules, or to assist with the immense intracellular organization that occurs during the T cell response.

## Data Availability Statement

The raw data supporting the conclusions of this article will be made available by the authors, without undue reservation.

## Ethics Statement

The animal study was reviewed and approved by the Duke University IACUC.

## Author Contributions

IM designed experiments, wrote the manuscript, crossed the mice, and analyzed the data. ZC performed experiments and analyzed the data. RS performed the experiments and wrote the manuscript. Y-WH designed the experiments, analyzed the data, and wrote the manuscript. All authors contributed to the article and approved the submitted version.

## Conflict of Interest

The authors declare that the research was conducted in the absence of any commercial or financial relationships that could be construed as a potential conflict of interest.

## Publisher’s Note

All claims expressed in this article are solely those of the authors and do not necessarily represent those of their affiliated organizations, or those of the publisher, the editors and the reviewers. Any product that may be evaluated in this article, or claim that may be made by its manufacturer, is not guaranteed or endorsed by the publisher.

## Abbreviations

AVO: Acidic vesicular organelle
Inpp4: inositol polyphosphate 4-phosphatase
LC3: Microtubule associated protein light chain 3
PI3K: Phosphatidylinositol 3 Kinase
PI(3)P: Phosphatidylinositol 3-phosphate
SHIP: SH2 domain-containing inositol 5′-phosphatase
TCR: T cell receptor.
