# Supplementary material for: Class I PI3K Provide Lipid Substrate in T Cell Autophagy Through Linked Activity of Inositol Phosphatases
Source: Front Cell Dev Biol. 2021 Aug 12;9:709398. doi: 10.3389/fcell.2021.709398 (PMC8397451; doi:10.3389/fcell.2021.709398)
Supplement: Supplementary file 1 [file Data_Sheet_1.docx]

**­
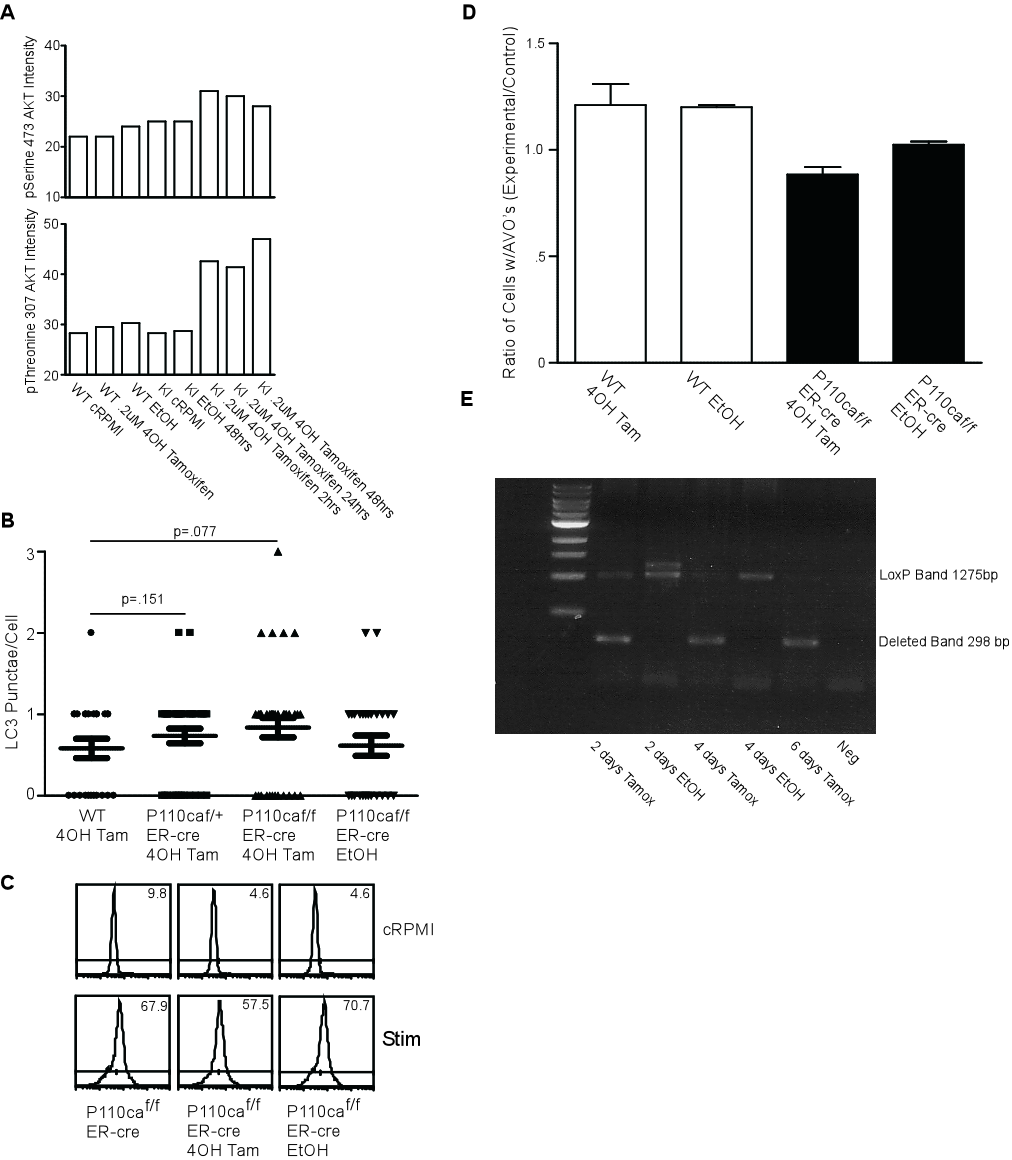
**

**Supplemental Figure 1.** (A) pAKT Western blot intensities for constitutively active P110α knock in CD4 T cells (KI=P110ca^f/f^ER-cre). CD4 T cells were subjected to 500nM 4OH Tamoxifen treatment for the indicated times and protein isolated for Western Blot. (B) LC3 punctate formation in resting naïve CD4 T cells expressing 0, 1, or 2 alleles of constitutively active p110α, 48 hours post induction with 500nM 4OH Tamoxifen. (C) Representative histograms detailing AVO formation of CD4 T cells treated as in (B) and starved for 48 hours in HBSS (D) Quantitation of (C), representative of 4 independent experiments. (E) Genomic deletion efficiency of p85 in p85^f/f^ER-cre CD4 T cells. CD4 T cells were treated with 4OH Tamoxifen for the indicated times and DNA was isolated. 2 bands corresponding to the deleted band (298bp) and the loxP band (1275bp) were visualized. By 4 and 6 days, all the loxP flanked genomic DNA was deleted by 4OH Tamoxifen.

**
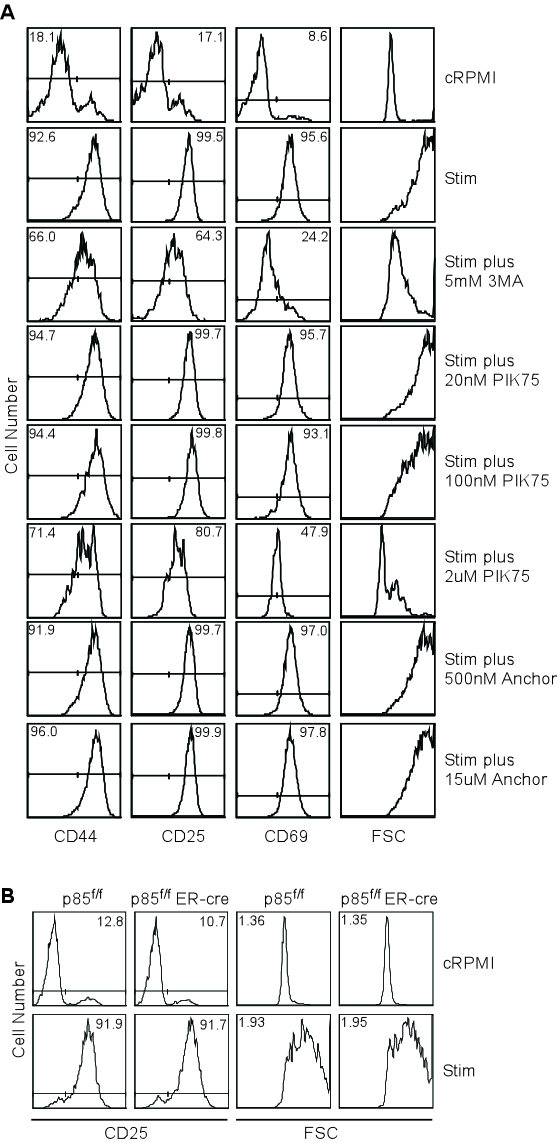
**

**C**


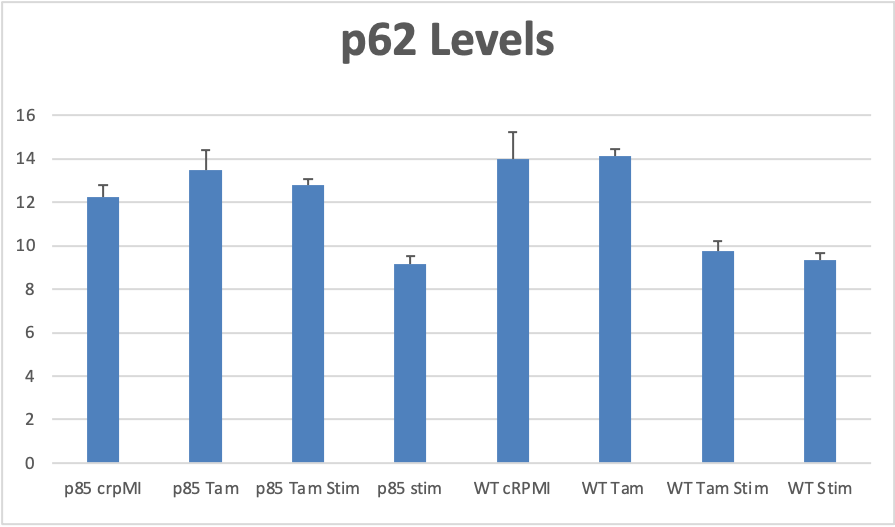


p=.04

p=.006

**D**


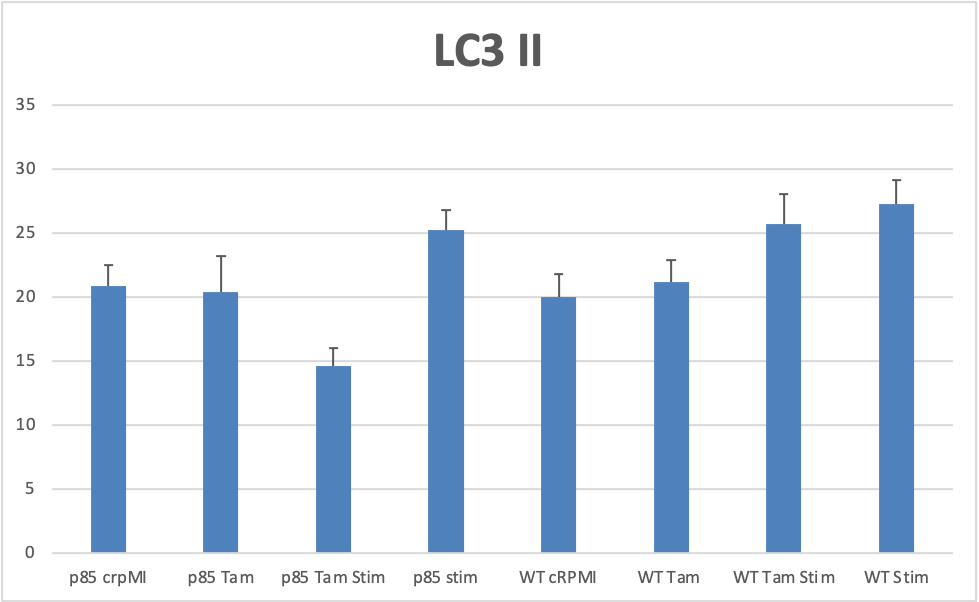


p=.002

p=.030

**Supplemental Figure 2.** (A) Activation of T cells under PI3K inhibitory conditions. Total splenocytes were activated with soluble anti-CD3 and anti-CD28 for 48 hours in the presence of the indicated inhibitors and upregulation of CD25, CD44, and CD69 measured. Additionally, forward scatter (FSC) was used as a measure of cell growth into blasts. (B) p85^f/f^ER-cre and p85^f/f^ splenocytes were treated with tamoxifen for 92 hours and stimulated with anti-CD3 and anti-CD28 for 48 hours, and CD25 and FSC measured. (C) Quantification of p62 degradation from main paper Figure 2E. Deletion of p85/p55 results in reduced p62 degradation, whereas TCR-stimulation leads to degradation of p62. (D) Quantification of LC3 II formation from main paper Figure 2E. Deletion of p85/p55 results in reduced LC3 II formation, whereas TCR stimulation leads to LC3 II formation

**
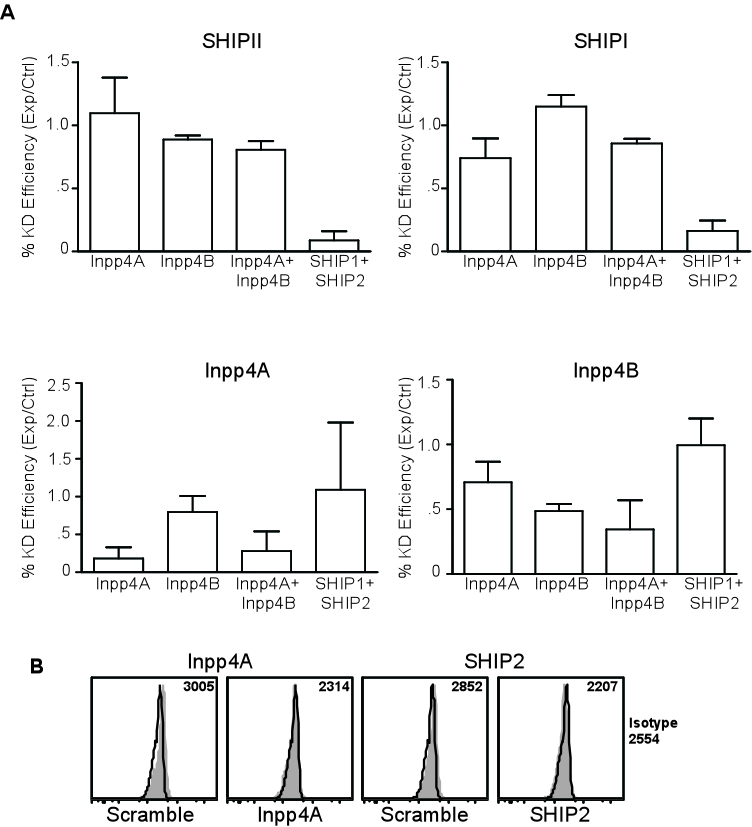
**

**Supplemental Figure 3.** (A) QPCR of Inpp4A, Inpp4B, SHIP1, and SHIP2 under the indicated siRNA cocktails. mRNA was isolated 72 hours after electroporation of 5ug/5x10^6^ cells with siRNA clones. Data were normalized to β-actin and relative knockdown levels were calculated compared to a scrambled control siRNA. (B) Protein expression levels of Inpp4A and SHIP2. hPBMCs were electroporated with siRNA to Inpp4A and SHIP2 and allowed to recover for 72 hours. Cells were then stimulated with .5ug/mL anti-CD3 and anti-CD28 for 48 hours, permeabilized, and stained for Inpp4A and SHIP2. Shaded histograms indicate protein, Unshaded histograms represent isotype controls, and numbers indicate mean fluorescence of proteins. Data are representative of 3 donors in 2 independent experiments.

**
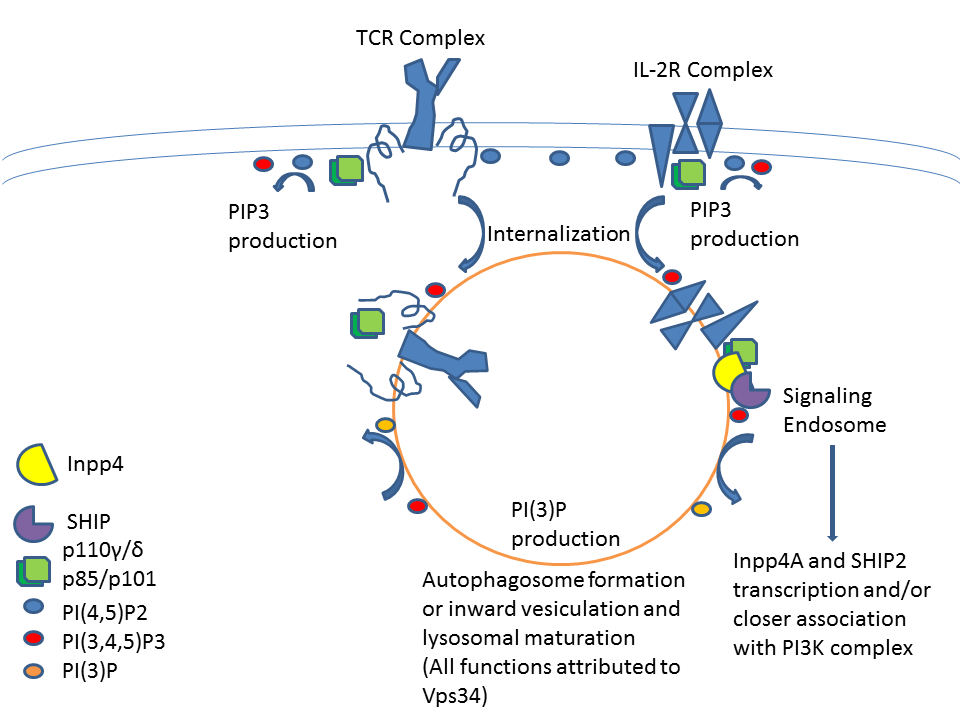
**

**Supplemental Figure 4.** Model of linked activity of class I PI3K and inositol phosphatases in TCR mediated autophagy. TCR signals activate the PI3K pathway, specifically P110γ and P110δ. PI(3,4,5)P3 is produced on the inner leaflet of the plasma membrane to facilitate the activation of Akt to support the cell’s energy requirements. However, as the TCR complex is internalized, PI(3,4,5)P3 is continuously produced on endomembranes. Meanwhile, Inpp4 and SHIP transcriptional levels are enhanced leading to greater inositol phosphatase levels and/or tighter association with the PI3K complex, resulting in the production of PI(3)P. This leads to the recruitment of proteins that promote autophagy, as well as inward traffic.
